# The effect of chemical and structural modifiers on the haemostatic process and cytotoxicity of the beta-chitin patch

**DOI:** 10.1038/s41598-021-97781-8

**Published:** 2021-09-17

**Authors:** Ahad Sabab, Sha Liu, Shari Javadiyan, C. John McAdam, Lyall R. Hanton, Alistair Jukes, Sarah Vreugde, Peter-John Wormald

**Affiliations:** 1 Department of Surgery-Otorhinolaryngology, Head and Neck Surgery, University of Adelaide, Adelaide, Australia; 2 Department of Chemistry, University of Otago, Dunedin, New Zealand

**Keywords:** Biotechnology, Health care, Medical research

## Abstract

Beta-chitin patch has previously been proven to be an effective haemostat, but whether modifying the patch affects its efficacy and safety, remains unanswered. In this study, the patch was modified using polyethylene oxide, Pluronic-F127, calcium, increased thickness or polyphosphate, and their effect on the process of haemostasis and cytotoxicity was tested and compared with standard-of-care, Surgicel and FloSeal. Whole blood collected from volunteers was applied to the patches to test their whole blood clotting and thrombin generation capacities, whilst platelet isolates were used to test their platelet aggregation ability. The fluid absorption capacity of the patches was tested using simulated body fluid. Cytotoxicity of the patches was tested using AlamarBlue assays and PC12 cells and the results were compared with the standard-of-care. In this study, beta-chitin patch modifications failed to improve its whole blood clotting, platelet aggregation and thrombin generation capacity. Compared to non-modified patch, modifications with polyethylene oxide or calcium reduced platelet aggregation and thrombin generation capacity, while increasing the thickness or adding polyphosphate decreased platelet aggregation capacity. The cytotoxicity assays demonstrated that the beta-chitin patches were non-toxic to cells. In vivo research is required to evaluate the safety and efficacy of the beta-chitin patches in a clinical setting.

## Introduction

Intraoperative bleeding is the leading cause of death during surgery^1^. It is particularly concerning in the endoscopic and neurosurgical setting where intraoperative bleeds can distort the surgeon’s visual field^1^ and increase the risk of post-operative haematoma^2^. This can affect the outcome of the patient following surgery^2^.

There currently exist a variety of haemostatic techniques and agents that can be utilised to manage bleeds during surgery^3, 4^. Haemostatic modalities such as mechanical compression or electro-cautery are two of the oldest techniques used to manage haemorrhage^4^. While they effectively manage large vessel bleeds^4^, they provide inadequate management of a diffuse tissue ooze^5^. Furthermore, both mechanical compression and electro-cautery carry the risk of compressing or thermally injuring the surrounding tissue (especially neural tissue) and completely occluding the blood vessels^5^.

Haemostatic agents are now commonly used for managing intraoperative bleeds during surgery^3^. Currently there are several Food and Drug Administration (FDA) approved haemostats, with gelatine-thrombin matrix sealants (FloSeal) and oxidised regenerated cellulose (Surgicel) being the two most popular haemostats in surgery^3, 4^. In specialized surgical situations such as during neurosurgery both FloSeal and Surgicel have an intrinsic tendency to absorb fluids and swell^6, 7^. This can compress the surrounding neural tissue and has a detrimental impact on the neurosurgical outcome^6^.

Chitin is a naturally occurring biopolymer composed of N-acetyl glucosamides that can be found in the exoskeleton of arthropods and cell walls of fungi. The biological source determines the spatial arrangement of the polyglucosamide chains. Specifically, the forms commonly encountered are alpha-chitin with anti-parallel chain arrangement, beta-chitin with parallel alignment and gamma-chitin with a combination of parallel and anti-parallel arrangement^8^. Deacetylation of the glucosamide carbohydrate chain, typically with a strong base, generates the polyglucosamine derivative chitosan.

Research has found both chitin and chitosan to possess antimicrobial^9, 10^, antiadhesive^11, 12^, wound healing^13, 14^ and haemostatic properties^15, 16^, making them ideal for biomedical applications.

Whilst there exists an abundance of literature on alpha- and beta-chitin, literature on the properties of gamma-chitin and its bio-technological potential is currently lacking. For this reason, gamma-chitin was not considered for this research. Studies on alpha- and beta-chitin has demonstrated both to have excellent haemostatic properties. However, research by Smith et al. has demonstrated beta-chitin has a superior haemostatic potential compared to alpha-chitin and chitosan^17^.

The haemostatic ability of chitin and chitosan is derived from that of their glucosamide (GlcNAc) and glucosamine subunits. Poly-*N*-acetyl glucosamide (p-GlcNAc) exerts its haemostatic effects through a myriad of pathways, which include erythrocyte aggregation^18^, platelet aggregation^19^ and vasoconstriction^16^. Additionally, platelets are activated on contact with p-GlcNAc, forming platelet plugs^20^. Furthermore, the erythrocytes attached to the p-GlcNAc uptake the surrounding nitric oxide (NO)^16^, which is a vasodilator, whilst activated platelets release thromboxane^21^, which is a vasoconstrictor. The scarcity of factors that induce vasodilatation and abundance of factors causing vasoconstriction results in overall vasoconstriction^16, 21^.

In recent years, our team, in conjunction with The University of Otago, have developed a solid patch of beta-chitin. The toxicity of this beta-chitin patch on cells and their effect on the haemostatic process with and without structural or chemical modifiers is currently unknown.

In this study, the beta-chitin patch was modified with polyethylene oxide (PEO), Pluronic-F127, calcium, increased thickness or polyphosphate, followed by evaluation of its haemostatic ability and cytotoxicity in vitro. PEO has been found to improve the flexibility and sealing ability of the chitosan membrane, allowing it to better adhere to the site of injury; thus by modifying the beta-chitin patch with PEO, similar levels of flexibility and sealing ability are expected^22^. Surfactants are known to ensue better dispersion of nanofibers, therefore, adding Pluronic-F127 to the beta-chitin patch, should allow the chitin fibres to disperse more evenly throughout the patch, instead of aggregating in select areas of the patch^23^. Calcium and polyphosphate are a key activators of the coagulation cascade, therefore, the goal of modifying beta-chitin patch with either calcium or polyphosphate is to enhance coagulation in the vicinity of the patch, at the site of the injury^29^. Whereas increasing the thickness of the patch is expected to enhance erythrocyte and platelet trapping, resulting in a larger and more stable clot formation.

The aim of this paper is to study the effect of beta-chitin patch modification on the haemostatic process and cytotoxicity in vitro.

## Methods

### Beta-chitin patch preparation

#### Chitin digestion and dispersion

Dry squid pens were ground and the fraction that passed through a 250 μm sieve (Endecotts) collected. Protein was digested using 1 M NaOH (40 °C, 48 h) and the resulting solid collected by filtration, rinsed with H_2_O till pH neutral, then ethanol and air-dried. Typical weight loss for the digestion process was 53%.

The degree of acetylation (DA) was calculated using the equation proposed by Xu et al.^25^: = [(C/N − 5.14)/1.72] × 100. The crystalline index (CrI_010_) was determined using the equation developed by Focher and Zhang research groups^26, 27^. Thus: CrI_010_ = (*I*_010 _− *I*_am_) × 100/*I*_010_; where *I*_010_ and *I*_am_ are the intensities at 8.1° and 15° 2θ respectively.

The digested chitin was suspended in acetic acid (1% v/v) and dispersed by a mechanical blender to give ‘Chitin’, an opaque thick suspension with no separate liquid.

#### Bilayer patch fabrication

All backings were prepared by filtering with suction a portion of dispersed Chitin/PEO 1000 (20 wt% of chitin) on Whatman paper to dryness. Typical backing layer dimensions: ø 42 mm × 0.01 mm, weight 26 mg (~ 5% PEO by wt). For the foam topping (Table 1), 5.0 g of the selected chitin dispersion [one of Chitin, Chitin/PEO 1000 (20 wt% of chitin), Chitin/Pluronic F127 (20 wt% of chitin), Chitin/calcium acetate monohydrate [5743-26-0] (5, 10, 20 wt% of chitin), or Chitin/sodium phosphate glass (Aldrich S4379, 20 wt% of chitin)] was added to a backing layer disk in a petri dish, the combination frozen then lyophilised for > 24 h. Typical bilayer patch dimensions: ø 39 mm × 2 mm, 50–55 mg. The nominal calcium loadings were 0.3, 0.6 and 1.1 mg Ca^2+^ per patch for 5, 10 and 20 wt% preparations respectively.

**Table 1:** Chitin patch composition.

Patch descriptor	Foam composition (wt %)
Chitin	100% chitin
Chi/F127	83% chitin + 17% pluronic F127
Chi/PEO	83% chitin + 17% PEO 1000
Chi/5%Ca	95% chitin + 5% calcium acetate monohydrate
Chi/10%Ca	91% chitin + 9% calcium acetate monohydrate
Chi/20%Ca	83% chitin + 17% calcium acetate monohydrate
Chi/Thick	100% chitin (double thickness)
Chi/PP	83% chitin + 17% sodium phosphate glass

#### Thick bilayer patch fabrication

**Figure 1:**
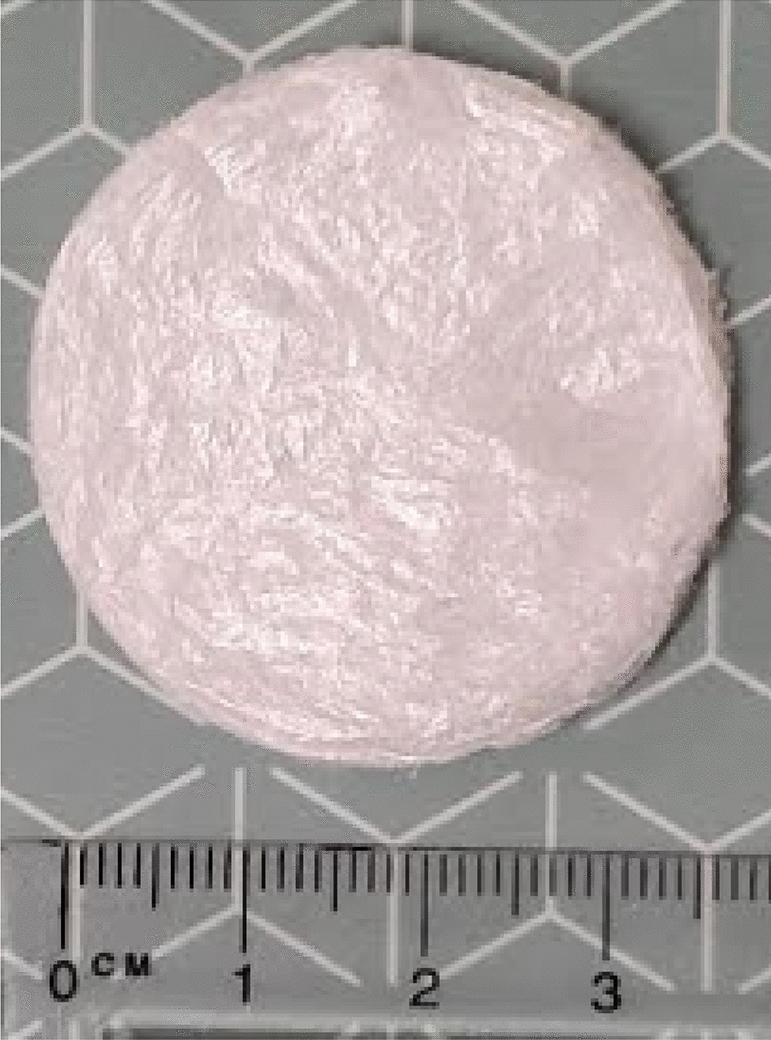
Beta-chitin patch following preparation.

Thick bilayer patches were prepared similarly from 10.0 g of the Chitin dispersion (*foam topping*). Typical thick patch dimensions: ø 39 mm × 4–5 mm, 100 mg (Fig. 1).

Following the preparation of the beta-chitin patches, the haemostats were gamma irradiated to ensure sterility.

### Ethics

Prior to commencement of this study, ethics approval was sought and granted by the Central Adelaide Local Health Network Human Research Ethics Committee (Ethics Approval #: HREC-15-TQEH-132). All experiments in this study were undertaken in accordance with the ethics approval and organisational policies and guidelines.

### Whole blood clotting experiment

This experiment was adapted from Shih et al.^28^. Whole blood was collected from five patients, following informed consent, at The Queen Elizabeth Hospital using EDTA (ethylenediaminetetraacetic acid) tubes (Pacific Laboratory Products, Victoria, Australia), to prevent coagulation. Exclusion criteria were haematological disorders and the use of antibiotics or anticoagulants in the 4 weeks prior to blood donation. The beta-chitin patches were cut to the dimensions of 1 cm × 1 cm and 0.20 mL of whole blood, followed by 0.02 mL of 0.2 M CaCl_2_, were added to the patches at room temperature. The samples were then incubated at 37 °C for 10 min. Following incubation, 10 mL of deionised water was added to lyse all erythrocytes not attached to the patches, releasing haemoglobin into the sample. 10 mL of the haemoglobin sample was collected and incubated for a further 60 min at 37 °C, after which the absorbance was measured at 540 nm.

### Platelet isolation

To isolate platelets, blood samples collected from patients were first centrifuged at 180×*g*, for 20 min. This was done to separate erythrocytes from platelet rich plasma. Following centrifugation, the supernatant (platelet rich plasma) was collected and further centrifuged at 1500×*g* for 15 min, to isolate the platelet pellet and platelet poor plasma. The platelet pellet was then resuspended in buffer consisting of 140 mM NaCl, 3 mM KCl, 12 mM NaHCO_3_, 0.4 mM NaH_2_PO_4_, 0.1% glucose and pH adjusted to 7.4 with 4% HEPES (4-(2-hydroxyethyl)-1-piperazineethanesulfonic acid), as previously described by Ong et al.^29^. The number of platelets in the suspension was quantified using a haemocytometer and only suspensions containing > 100,000 platelets/mL were used in the platelet aggregation experiment.

### Platelet aggregation experiment

The platelet aggregation experiment was adapted from Ong et al.^29^. Prior to starting this experiment, resuspended platelets were reconstituted to 2.5 mM CaCl_2_ and 1.0 mM MgCl_2_. 1.2 mL of platelet suspension was added to each patch and the sample was incubated for 60 min at 37 °C using a water bath. Following incubation, the patches were dip-rinsed twice in PBS (phosphate buffer solution) to remove all unattached platelets. The sample was then placed in PBS containing 0.9% Triton-X100 and further incubated for another 60 min at 37 °C. For control, 1.2 mL of platelet isolates were placed in PBS containing either 0.9% Triton-X100 (negative control) or 10% Triton-X100 (positive control) and incubated for 60 min at 37 °C. The Triton-X100 caused lysis of all platelets attached to the patch, releasing lactate dehydrogenase (LDH) enzymes into the sample. The LDH levels within the sample were analysed using an LDH assay kit (Promega, New South Wales, Australia), as per manufacturer’s instructions.

### Thrombin generation assay

The patches were cut to dimensions of 1 cm × 1 cm and they were immersed in 1 mL of heparinised whole blood. Following 10-min incubation at 37 °C, 20 μL of 0.633 M of sodium citrate was added to the sample to stop thrombin generation. The sample was centrifuged at 200×*g* for 10 min, after which the plasma was collected and the thrombin level was measured using Thrombin-Anti-thrombin Assay kit (Abcam, Cambridge, USA), as per the manufacturer’s instructions.

### Fluid absorption capacity of chitosan patches

Simulated body fluid (SBF) was used to assess the absorptive capacity of the beta-chitin patches, gauze and Surgicel. Since FloSeal is a gel, direct analysis of its absorptive capacity was not possible. However, given that FloSeal is composed of thrombin and gelatin, with gelatin being the absorptive material within the haemostat, FloSeal was substituted with Gelfoam, a dry, solid, foam-like composition of gelatin. SBF was formulated as described by Kokubo et al.^30^. Beta-chitin patches of dimension 1 cm × 1 cm were weighed (W_i_) and placed in 24-well tissue culture plates. 2 mL of SBF was added to each well and the plate incubated for 48 h at 37 °C. Following incubation, the patches were re-weighed (W_f_) and fluid uptake (FU) per cm^2^ was calculated as per the formula, FU = (W_f_ − W_i_)/1 cm^2^.

### Tissue adhesion study

This experiment was adapted from Castro et al.^31^. The beta-chitin patches and Surgicel were cut to dimensions of 1 cm × 1 cm and compressed at 20 kPa to the centre of individual stripes of bovine muscle tissue. The patches were held for 3 min after which the bovine tissue was fixed taut on two opposite ends vice grips, with the dressing on the bottom side. Hanging weights (0.01 N) were sequentially attached to the patches and Surgicel until they detached from the tissue. The weight at which the patches/surgical detached was used to calculate the adhesion strength (kPa). FloSeal was not tested in this experiment since its gel-like nature impairs its adhesive properties.

### Scanning electron microscopic examination

To compare the physical structure of the beta-chitin patches, Surgicel and FloSeal, they were mounted on aluminium stubs (Proscitech), sputter coated with 5 nm platinum and examined in a Philips XL 30 FESEM scanning electron microscope.

### Cytotoxicity assay

For this experiment, a PC-12 cell line was kindly donated by the Discipline of Pharmacology, University of Adelaide. The PC-12 cell line is a cell line of neural origin, derived from rat pheochromocytoma^32^. PC-12 cells were cultured in Dulbecco’s Modified Eagle Medium (DMEM) with 4.5 g/L d-Glucose, l-Glutamine and 25 mM HEPES (Gibco, Victoria, Australia), supplemented with foetal bovine serum, L-glutamate and penicillin and streptomycin. 1 × 10^4^ PC-12 cells were seeded into each well of a 96-well plate and incubated overnight at 37 °C in a 5% CO_2_ in air atmosphere.

Modified and non-modified beta-chitin patches, Surgicel and FloSeal extracts were formulated by immersing the haemostats in supplemented DMEM media at a concentration of 10 mg/mL. The whole sample was incubated overnight at 37 °C. Following overnight incubation, the media was removed from the PC-12 cell culture and cells were treated with modified or non-modified beta-chitin patch, Surgicel or FloSeal extract, culture media or 10% Triton X-100 in PBS. The cells were then further incubated at 37 °C for 24 h and 48 h. Following incubation, the culture media was removed and 100 μL of supplemented DMEM media comprising 10% AlamarBlue (Life Technologies, Victoria, Australia) was applied to the cell culture. Fluorescence was measured at 4-h using 530 nm excitation and 590 nm emission. The raw data was analysed to evaluate cell viability.

### Statistical analysis

All data is expressed as mean ± standard deviation. Kruskal–Wallis one-way ANOVA was utilized for statistical analysis; using Dunn’s test with Benjamini–Hochberg correction to perform pair-wise comparisons. p values < 0.05 were considered statistically significant.

## Results

### Beta-chitin patch preparation and characterisation

The patches were prepared from beta-chitin from the New Zealand Arrow squid (*Notodarus sloanii*). The pens provide a low ash chitin source typical of squid species^33–35^. Protein was removed by digestion with warm 1 M sodium hydroxide. These mild alkaline conditions minimise deacetylation of the poly *N*-glucosamide carbohydrate chains. [This contrasts with the conditions for preparation of chitosan, which aims for deacetylation to the polyglucosamine form and typically employs ≥ 12 M NaOH at elevated temperatures^27, 36^.] Due to the low levels of ash an acidic demineralisation was not deemed necessary.

**Figure 2:**
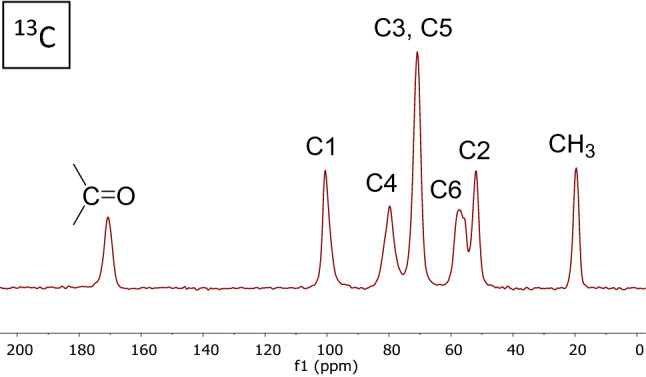
^13^C solid-state spectrum of digested chitin.

Elemental analysis (Table 2) gives a C/N ratio of 4.0 for the protein-rich raw squid pens, and for the digested material this ratio rises to 6.6. The degree of acetylation (DA) was calculated using the equation proposed by Xu et al*.*^25^. This gives a value of 86.5% confirming minimal deacetylation. Solid state ^13^C NMR (Fig. 2) was also used to provide a value of acetylation by comparing the intensity of the acetyl CH_3_ resonance with the sum of the polyglucosamide ring resonances^37^. This method gives a DA value of 81% for the digested squid pen chitin. Thermogravimetric analysis (TGA) shows 10% of the digested chitin weight is bound water.

**Table 2:** Raw and digested chitin.

	Squid pen	Chitin
C (%)	47.1	42.2
H (%)	7.18	7.20
N (%)	11.9	6.36
C/N	4.0	6.63
Degree acetylation (%)		86.5
Ash (%)	1.3	0.5
Moisture (wt %)		10

**Figure 3:**
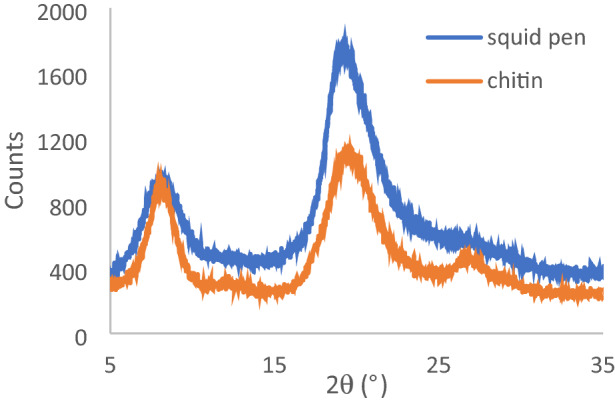
X-ray diffraction pattern for raw and digested chitin.

The X-ray diffraction pattern of the digested squid pen chitin is shown in Fig. 3, and for comparison purposes that of the undigested squid pens. The position of the maxima are known to be dependent on the degree of hydration and in this case though not well-resolved show no shift during the digestion process. The crystalline index (CrI_010_) was determined as 70% using equations developed for alpha-chitin by the Focher and Zhang research groups^26, 27^. The diffraction pattern and crystalline index match closely those reported for the Argentinian short-finned squid *Ilex argentines*^34^.

**Figure 4:**
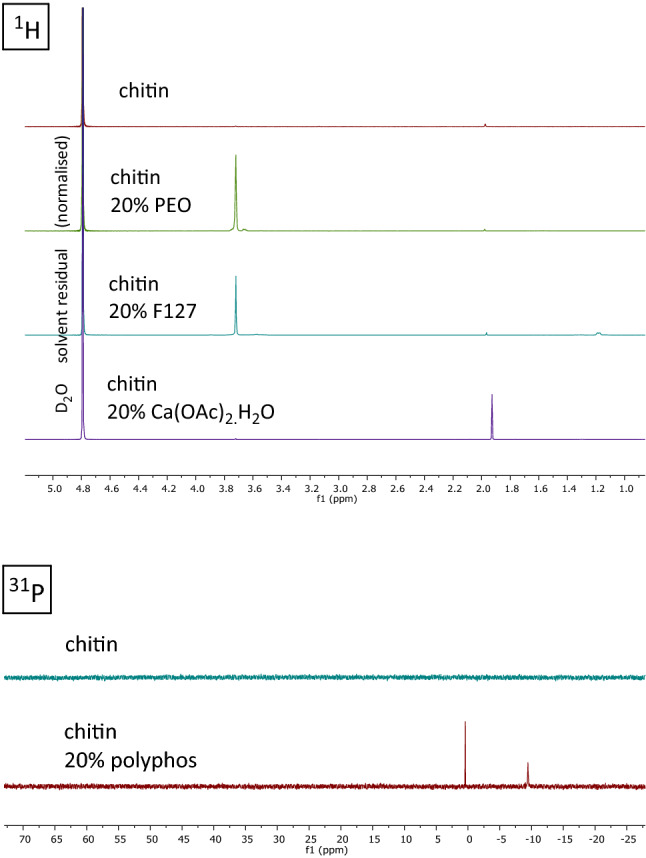
Additive release from patches: ^1^H and ^31^P NMR in D_2_O. For experimental details see Supplementary Material.

The patches comprised a bilayer system from a suspension of the chitin in 1% acetic acid. To provide some mechanical stability an initial backing was formed by filtration to dryness of a portion of chitin suspension. On top of this was placed the equivalent weight of suspension, and the whole rapidly frozen. Lyophilisation produced a patch of light foam topping (approx. 2 mm thick) on 0.01 mm chitin backing. Modified patches were prepared by incorporation of polyethylene oxide, the FDA approved poloxamer surfactant Pluronic F127, calcium ions (using calcium acetate monohydrate) and polyphosphate (a sodium glass). The additives at the desired loading were blended to homogeneity in the chitin suspension to provide even distribution in the foam. The additives were non-volatile, totally soluble and there was no evidence of separation/partition during the lyophilisation process, see SEM images (Fig. 10). The additives are not chemically bonded to the chitin matrix, and evidence for rapid and near-quantitative release/availability was provided by ^1^H NMR and ^31^P NMR studies (Fig. 4; Supplementary Material). Additional characterisation using FTIR, notes on methodology, and selected mineral content from ICPMS analyses are provided in Supplementary Material.

### Whole blood clotting

**Figure 5:**
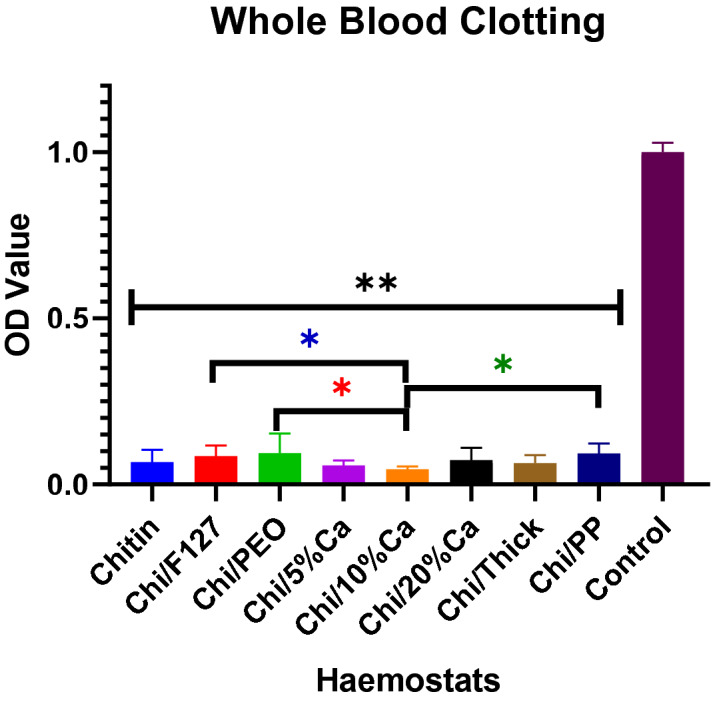
The effect of chemical and structural modification on whole blood clotting ability of the beta-chitin patch. The absorbance (OD value) of non-modified beta-chitin patch (Chitin), Chi/F127, Chi/PEO, Chi/5%Ca, Chi/10%Ca, Chi/20%Ca, Chi/Thick and Chi/PP are normalised to negative control. Lower absorbance indicates higher clotting. **p < 0.001 means significantly higher compared to the modified and non-modified beta-chitin patches, *p < 0.05 means significantly lower compared to Chi/PEO, *p < 0.05 means significantly lower compared to Chi/F127 and *p < 0.05 means significantly lower compared to Chi/PP; analysed by Kruskal–Wallis one-way ANOVA with Dunn’s test, n = 5.

The whole blood clotting experiment measured the absorbance of haemoglobins released from erythrocytes that did not form a clot, when in contact with the patch. Therefore, the lower the absorbance (OD value), the greater the whole blood clotting within the patches. Compared to negative control, the non-modified beta-chitin patch and all modifications of that patch significantly reduced OD values. There were no significant differences between the non-modified beta-chitin patch and any of the modified beta-chitin patches. Chi/10%Ca had the lowest OD value and it was significantly lower than Chi/F127, Chi/PEO and Chi/PP (p < 0.05) (Fig. 5).

### Platelet aggregation

This experiment assessed the relative quantity of platelets attached to the patch, compared to controls. When the platelets are applied to the patch, more will attach to the patches with higher platelet aggregation ability. Hence, when these patches are immersed in Triton-X100, more platelets will undergo lysis, resulting in higher quantity of LDH being released into the assay, resulting in a higher OD value. Therefore, for this experiment, higher OD value would indicate greater platelet aggregation ability.

**Figure 6:**
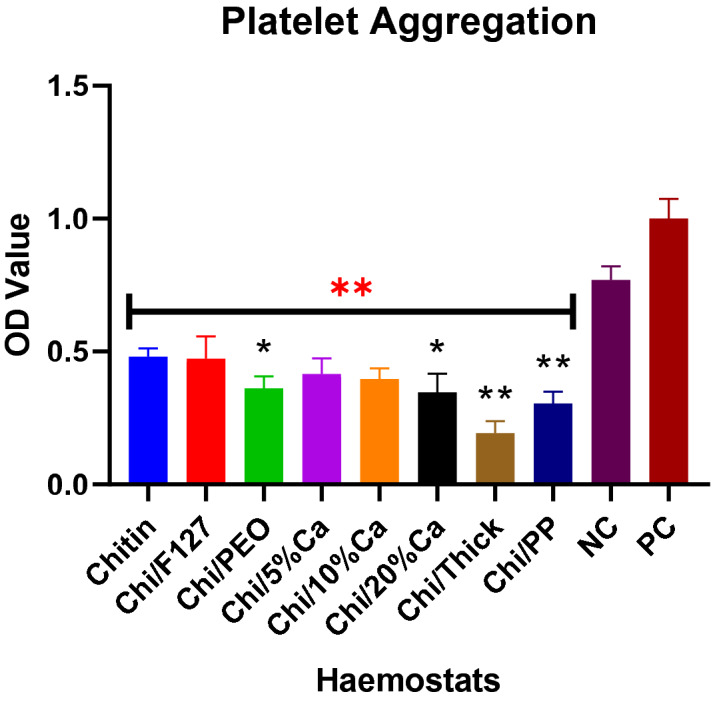
The effect of chemical and structural modification on the platelet aggregating ability of the beta-chitin patch, as measured by LDH assay. The OD value of non-modified beta-chitin patch (chitin), Chi/F127, Chi/PEO, Chi/5%Ca, Chi/10%Ca, Chi/20%Ca, Chi/Thick, Chi/PP and negative control (NC) are normalised to positive control (PC). *p < 0.05 and **p < 0.001, compared to non-modified beta-chitin patch and **p < 0.001, compared to positive (10% Triton-X100 in PBS) and negative (0.9% Triton-X100 in PBS) control, analysed by Kruskal–Wallis one-way ANOVA with Dunn’s test, n = 5.

In this experiment, compared to both positive and negative control, there was a significant reduction in OD value for the non-modified and all modified beta-chitin patches. From all chitin patches, the non-modified beta-chitin patch and Chi/F127 had the highest OD value compared to all other groups, indicating greater platelet aggregation ability (Fig. 6). Chi/PEO, Chi/20%Ca, Chi/Thick and Chi/PP, had significantly lower platelet aggregating index compared to the non-modified beta-chitin patch (p < 0.05). Chi/Thick had the lowest platelet aggregation index and, with the exception of Chi/PP (p = 0.28), it was significantly lower than all other beta-chitin patches (p < 0.001). Furthermore, increasing the concentration of calcium within the beta-chitin patches resulted in a subsequent reduction in the platelet aggregation index, but these changes were not statistically significant (Fig. 6).

### Thrombin generation

**Figure 7:**
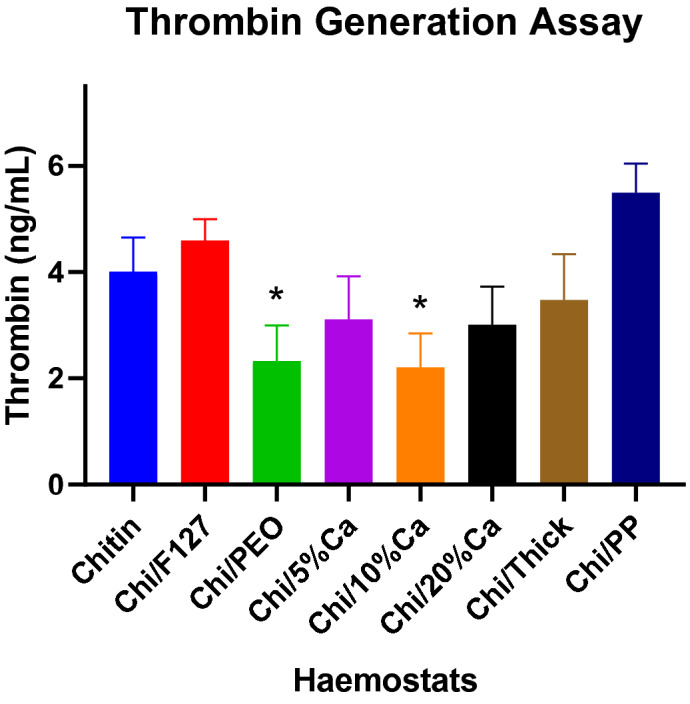
The effect of chemical and structural modification on the thrombin generating ability of the beta-chitin patch, as measured by thrombin–antithrombin assay (ng/mL). *p < 0.05 compared to non-modified beta-chitin patch, analysed by Kruskal–Wallis one-way ANOVA with Dunn’s test, n = 5.

Chi/PP generated the greatest amount of thrombin, compared to all other patches (Fig. 7). This was significantly greater than Chi/thick, Chi/PEO and all of the calcium enriched patches (p < 0.05), but not compared to the non-modified beta-chitin patch. Chi/PEO and Chi/10%Ca both had a significantly lower thrombin generating ability compared to the non-modified beta-chitin patch (p < 0.05) (Fig. 7).

### Fluid absorption capacity of chitosan patches

**Figure 8:**
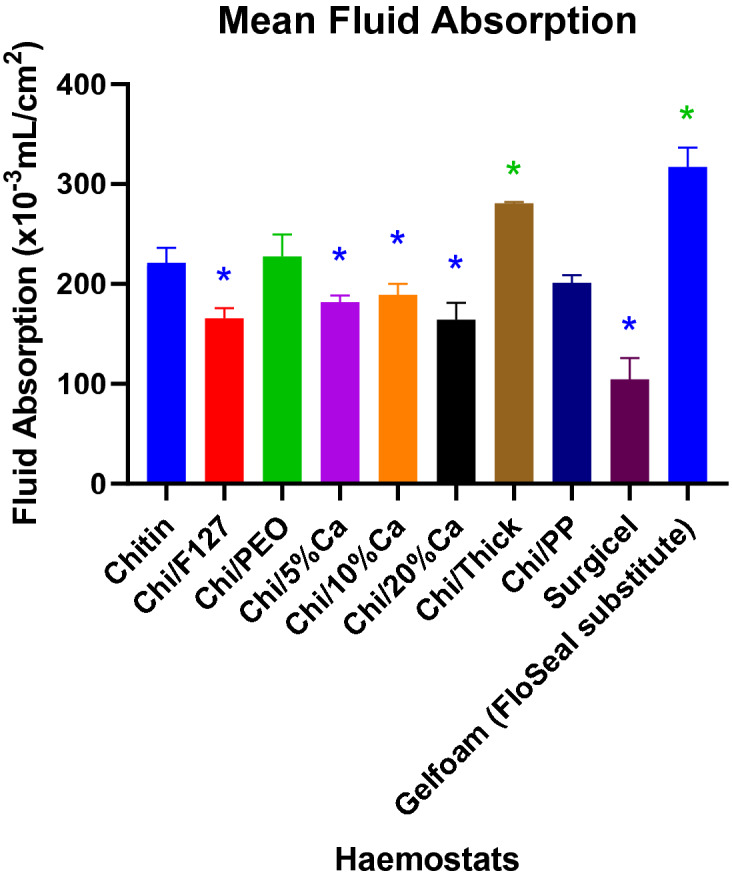
Fluid absorbed by modified and non-modified beta-chitin patches, Surgicel and FloSeal, per unit area (× 10^−3^ mL/cm^2^). *p < 0.05, significantly lower compared to non-modified beta-chitin patch. *p < 0.05, significantly higher compared to non-modified beta-chitin patch analysed by Kruskal–Wallis one-way ANOVA with Dunn’s test, n = 5.

Gelfoam, which is the FloSeal substitute, absorbed the most fluid per unit area, whilst Surgicel absorbed the least amount of fluid per unit area (Fig. 8). Chi/F127, Chi/5%Ca, Chi/10%Ca, Chi/20%Ca and Surgicel, all had significantly lower fluid absorption capacity compared to the non-modified beta-chitin patch (p < 0.05) (Fig. 8). Whereas, Chi/Thick and Gelfoam, both had significantly higher fluid absorption capacity compared to the non-modified beta-chitin (p < 0.05) (Fig. 8).

### Tissue adhesion study

**Figure 9:**
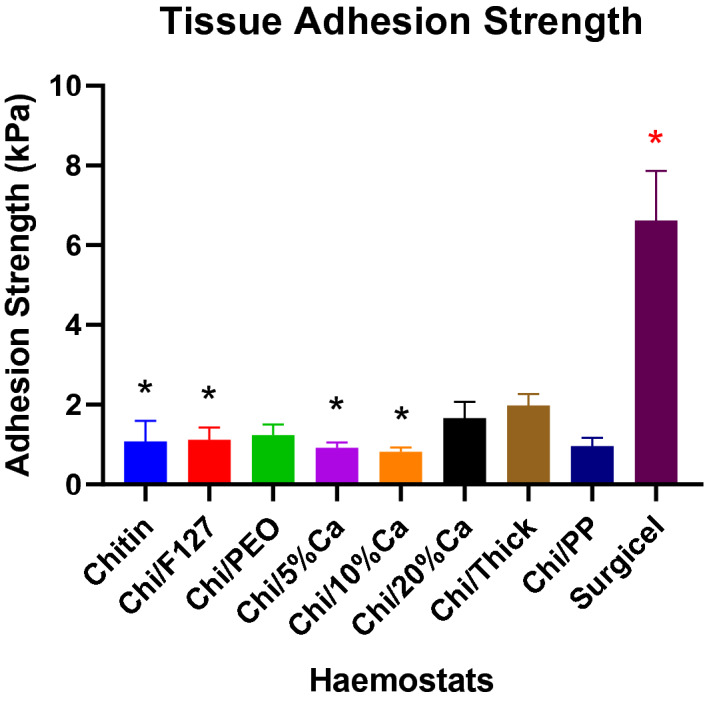
Tissue adhesion strength of modified and non-modified beta-chitin patches and Surgicel. *p < 0.05, significantly lower compared to Chi/Thick. *p < 0.05, significantly higher compared to modified and non-modified beta-chitin patch analysed by Kruskal–Wallis one-way ANOVA with Dunn’s test, n = 5.

Chi/Thick was the strongest adhesive compared to all other beta-chitin patches and its adhesion strength was significantly greater than non-modified beta-chitin patch, Chi/F127, Chi/5%Ca and Chi/10%Ca (p < 0.05) (Fig. 9). Furthermore, Surgicel had significantly stronger adhesion strength compared to all beta-chitin patches (p < 0.05) (Fig. 9).

### Scanning electron microscopic examination

**Figure 10:**
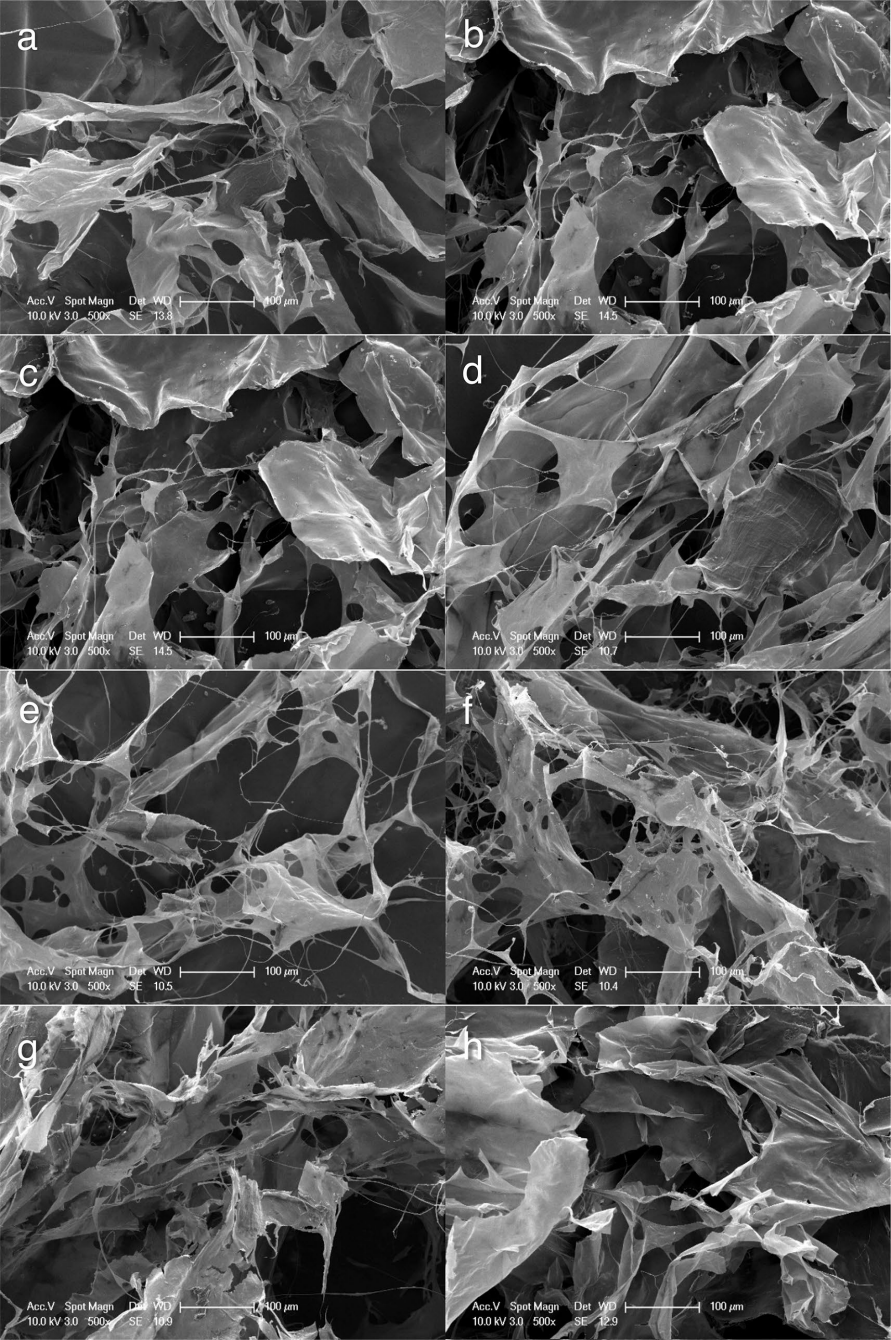
Scanning electron microscopy images of (**a**) non-modified beta-chitin patch, (**b**) Chi/F127, (**c**) Chi/PEO, (**d**) Chi/5%Ca, (**e**) Chi/10%Ca, (**f**) Chi/20%Ca, (**g**) Chi/Thick and (**h**) Chi/PP.

**Figure 11:**
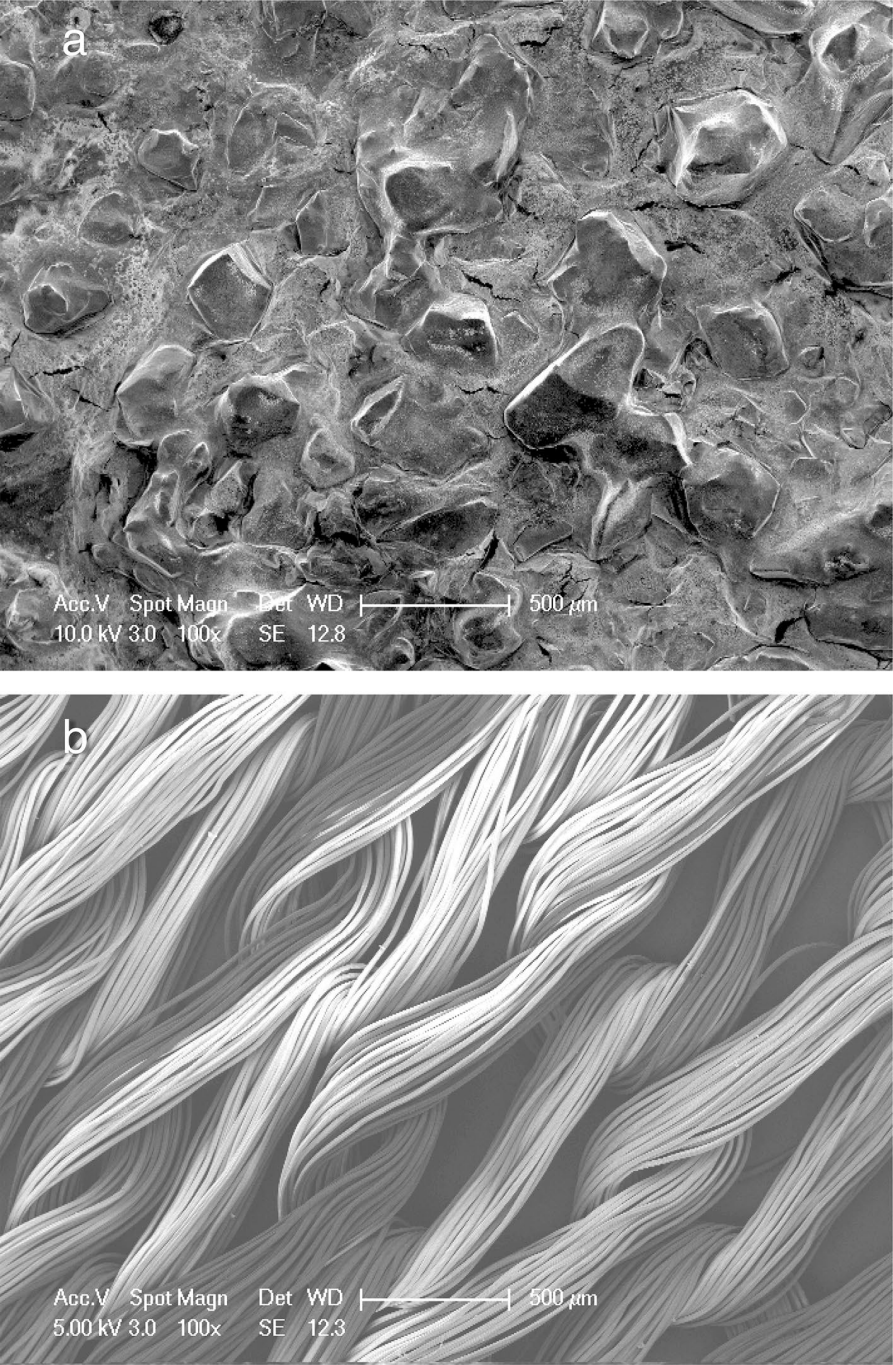
Scanning electron microscopy images of (**a**) FloSeal and (**b**) Surgicel.

The SEM images of the patches showed that in the beta-chitin patches, the fibres aggregate to form irregular layers (Fig. 10). Furthermore, there were no physical differences between the modified and non-modified beta-chitin patches (Fig. 10). SEM image of FloSeal showed the large gelatine component of its gelatine-thrombin matrix (Fig. 11). Whereas, the SEM image of Surgicel showed that individual fibrils coalesced to form Surgicel fibers, which in turn formed the mesh like structure (Fig. 11).

### Cytotoxicity assay

**Figure 12:**
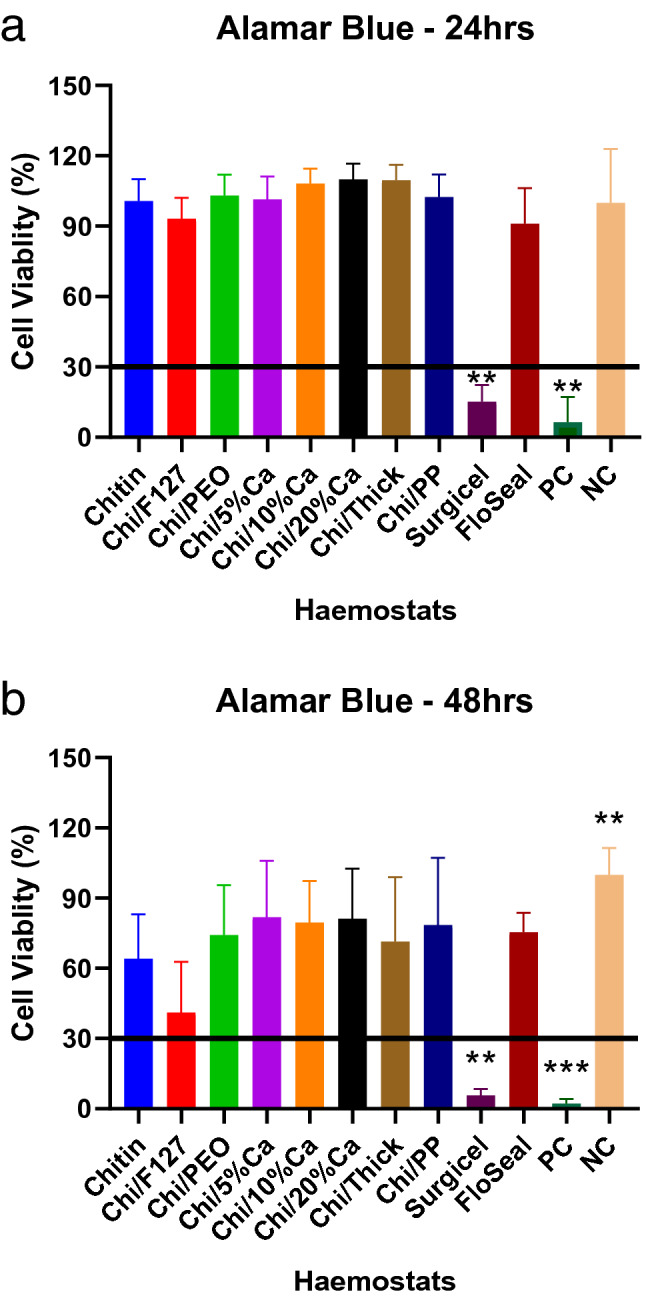
The cytotoxic effects of modified and non-modified beta-chitin patches, Surgicel and FloSeal on PC-12 cells as measured by AlamarBlue (**a**,**b**). AlamarBlue assay at (**a**) 24-h and (**b**) 48-h post-treatment, showing the cell viability with non-modified beta-chitin patch (Chitin), Chi/F127, Chi/PEO, Chi/5%Ca, Chi/10%Ca, Chi/20%Ca, Chi/Thick, Chi/PP, Surgicel, FloSeal and PC (10% Triton-X100 in PBS) treatments, normalised to NC (culture media). *p < 0.05 and **p < 0.001, compared to non-modified beta-chitin patch, analysed by Kruskal–Wallis one-way ANOVA with Dunn’s test, n = 5.

At 24 h, the beta-chitin patches and FloSeal had a cell viability of approximately 100% and were not different from the negative control (culture media) (Fig. 12). Whereas, the cell viability in the presence of Surgicel was approximately 15% and this was significantly lower than the non-modified beta-chitin patch group (p < 0.001). At 48 h, there was a decrease in the cell viability for all the haemostats and, similar to 24 h, the cell viability with Surgicel was significantly lower than the non-modified beta-chitin patch group (p < 0.001) (Fig. 12). There were no significant differences between the non-modified beta-chitin and FloSeal groups, at 24 or 48 h (Fig. 12).

## Discussion

In this study we investigate the effect of modified and non-modified beta-chitin patches on the haemostatic process and compare their cytotoxicity with current market leading haemostats. Our results show that specific modifications impart selective haemostatic advantages to the beta-chitin patches. In addition, regardless of the modification, none of the beta-chitin patches were cytotoxic to cells of neural origin and their safety was equal and superior to those of FloSeal and Surgicel, respectively.

In this study, three of the seven beta-chitin patches were calcium enriched patches. Calcium is a crucial component of haemostatic physiology. It not only influences the activation and aggregation of platelets, but calcium also plays a key role in activating coagulation factors^24, 38, 39^. In this study, the beta-chitin patches modified with varying concentrations of calcium demonstrated to have varying whole blood clotting and platelet aggregating abilities. Firstly, the whole blood clotting experiment demonstrated that modifying the beta-chitin patch with 10% calcium (Chi/10%Ca) significantly enhanced the whole blood clotting ability of the patch. Whilst this finding is in line with that reported by Sundaram et al., who demonstrated that chitosan hydrogen modified with calcium significantly improved its haemostatic potential, it fails to explain why there was a reduction in whole blood clotting with Chi/5%Ca and Chi/20%Ca^40^. A potential explanation could that found in a previous study by Jaques et al., who demonstrated that the prothrombin time (a measure of clotting rate) is optimised when the plasma calcium concentration is approximately 0.05 M and any variation from this optimal concentration resulted in a corresponding increase in prothrombin time^41^. Thus there is a possibility that when immersed in whole blood, Chi/10%Ca may have changed the surrounding extracellular calcium concentration to close to 0.05 M, whereas, Chi/5%Ca and Chi/20%Ca patches, might have respectively decreased or increased the extracellular calcium concentration beyond the optimal 0.05 M. Overall, this may have resulted in a significant increase in whole blood clotting with the Chi/10%Ca, but not Chi/5%Ca or Chi/20%Ca.

In addition, the platelet aggregation experiment further demonstrated that increasing the concentration of calcium within the beta-chitin patches resulted in a subsequent decrease in platelet aggregation. This could be because extracellular calcium significantly influences platelet aggregation, provided that the concentration remains within an optimal range^42^. However, when the calcium concentration exceeds the optimal range, as may be the case in this study, there is a subsequent reduction in platelet aggregation^42^.

In this study, Chi/PEO was noted to have significantly reduced platelet aggregation compared to the non-modified beta-chitin patches. PEO is a polyether with a wide range of use in medicine. While no direct relationship between PEO and platelet has been identified in literature, multiple studies have associated PEO with reduced platelet activation and aggregation^43–45^. This may potentially be the primary cause of reduced platelet aggregation within the Chi/PEO patch.

Polyphosphate is an anionic compound, found within all living organisms^46^. In a study by Ruiz et al. the authors demonstrated that platelets contain polyphosphate dense granules, which they secrete upon activation^47^. The polyphosphate then directly interacts with the coagulation cascade to accelerate coagulation factor activation and enhance fibrin clot structure^48–50^. However, there is currently no data in literature to suggest any coagulation promoting interaction between polyphosphate and platelets. In this study, Chi/PP demonstrated to have the lowest platelet aggregation but the highest thrombin generation capacity of all beta-chitin patches. The significantly reduced platelet aggregation capacity may be attributed to repelling electrostatic forces between the polyphosphate anions within the patch and the anionic surface of platelets, whilst the enhanced thrombin generation can be attributed to its ability to accelerate the coagulation cascade.

As mentioned previously, a major disadvantage of the current neurosurgical haemostats, including Surgicel and FloSeal, is that they swell upon contact with blood or tissue fluid^6, 7^, which in turn compresses the surrounding neural tissue^6^. The results from the fluid absorption experiment indicate that beta-chitin patches also have an intrinsic tendency to absorb fluid and swell in volume. However, the extent of fluid absorption and swelling varied depending on the modification adapted. Chi/F127, Chi/5%Ca^2+^, Chi/10%Ca^2+^ and Chi/20%Ca^2+^ had a significantly lower fluid absorption and swelling compared to the non-modified beta-chitin patch. Furthermore, the study demonstrated that the fluid absorptive capacity of Surgicel is approximately two-folds lower than the non-modified beta-chitin patch. This can be attributed to the gauze like structure of Surgicel, compared to almost foam like structure of the beta-chitin patch.

Along with haemostatic properties and low fluid absorptive properties, strong tissue adhesion is another crucial characteristic that haemostats must possess. By adhering strongly to the site of injury, haemostats can minimize blood loss and promote haemostasis. The result of the tissue adhesion strength demonstrated that both modified and non-modified beta-chitin patches adhere to tissue with similar strength, with Chi/Thick having, on average, the highest tissue adhesion strength. Furthermore, contrary to what is suggested in the literature, Chi/PEO did not have a superior adhesion strength compared to other beta-chitin patches. A potential reason for this finding is that, whilst the quantity of PEO used to modify the patch may be sufficient to improve its flexibility, it may not be adequate in quantity to improve tissue adhesion.

The cytotoxicity experiment in this study demonstrated that none of the beta-chitin patches were cytotoxic. However, whilst FloSeal demonstrated similar toxicity profile as the beta-chitin patches, Surgicel had a highly cytotoxic profile. This is because the cell viability in all treatment groups, with the exception of Surgicel, remained above the 30%. According to the International Organization for Standardization, treatments with cell viability below 30% are considered cytotoxic^51^.

The cytotoxic profile of Surgicel is well established in literature, with a study by Hexig et al. demonstrating that Surgicel is cytotoxic to V79 and L929 cell lines at varying levels of dilution^52^. This is thought to be because Surgicel lowers the pH within its vicinity^53^. While the acidic nature of the Surgicel gives it the antimicrobial ability, it can also have cytotoxic effects on cells^52^. The acidity of Surgicel was measured during the cytotoxicity experiment using the Surgicel extract and the pH recorded was 4.6 (result not shown).

Although the cytotoxicity experiments provided an indication of how cells of neural origin react to beta-chitin patches and commercial haemostats, it is not a perfect reflection of clinical scenarios. This is because when these haemostats are applied to living tissue, it can induce a foreign body reaction^54^ where inflammatory cells migrate to the region and release inflammatory markers^55^. These inflammatory markers can also damage cells^56^. Hence, in the context of applying these haemostats to living tissue, the clinical outcome will be dictated by both the direct toxicity of the patches on the tissue, as well as, by the inflammation induced by the haemostats.

Similarly, while the in vitro whole blood clotting, platelet aggregation and thrombin generation experiments demonstrated the different haemostatic advantages that each of the modifications can impart on the beta-chitin patch, these experiments cannot stimulate, nor predict with certainty, how the beta-chitin patches would behave in the setting of an haemorrhaging vessel, where dynamic variables such as the size of blood vessel, blood pressure and blood flow, may significantly influence the haemostatic activity of the beta-chitin patches.

For these reasons, in vivo research is required to assess the effect of the beta-chitin patches on living tissue and the effect of the beta-chitin patches in sealing a haemorrhage, using an animal injury and vessel injury model. Based on the results attained from the in vitro experiments above, it is expected that both modified and unmodified beta-chitin patches will be safe and will have similar haemostatic efficacy.

## Conclusion

Beta-chitin has previously been demonstrated to possess strong haemostatic capacity. Previous studies have furthermore shown that chemical or molecules such as polyethylene oxide, Pluronic-F127 and calcium have various effects on the flexibility and structure of chitosan-based products and might improve the overall haemostatic effectiveness of beta-chitin patches. This study investigated the effect of beta-chitin patch and its modifiers on the haemostatic process using whole blood clotting, platelet aggregation and thrombin generation experiments. Furthermore, the safety of the beta-chitin patches was evaluated and compared with the standards of care, Surgicel and FloSeal, using cytotoxicity studies. The results demonstrated that different beta-chitin modifiers were associated with variable abilities in whole blood clotting, platelet aggregation and thrombin generation. Chi/10%Ca had the best whole blood clotting ability, non-modified beta-chitin patch and Chi/F127 had the best platelet aggregating ability, whilst Chi/PP had the greatest thrombin generation ability. Furthermore, the cytotoxicity experiment demonstrated an absence of cytotoxicity for all beta-chitin products. There was a significant reduction of cell viability in the presence of Surgicel, indicative of its cytotoxic profile. Overall, these results demonstrate how the beta-chitin patch and its modifiers influence the haemostatic physiology, whilst maintaining an acceptable safety profile. However, further research entailing vascular and tissue injury models in animals is required to evaluate and compare the haemostatic ability, direct toxicity and immunogenicity of the beta-chitin patches and their modifiers on living tissue. This research will also help identify the best performing beta-chitin patch modifier, paving the way for future clinical use.

## Supplementary Information

Supplementary Information.

## Data Availability

All data generated during and/or analysed during the current study are available from the corresponding author on reasonable request.
