# Supplementary material for: The effect of chemical and structural modifiers on the haemostatic process and cytotoxicity of the beta-chitin patch
Source: Sci Rep. 2021 Sep 17;11:18577. doi: 10.1038/s41598-021-97781-8 (PMC8448852; doi:10.1038/s41598-021-97781-8)
Supplement: Supplementary file 1 — Supplementary Information. [file 41598_2021_97781_MOESM1_ESM.docx]

SUPPLEMENTARY MATERIAL

*Chitin and patch characterisation, instrumentation and methodology*

Elemental analysis and ashing were performed by the Campbell Microanalytical Laboratory, University of Otago. Trace element analysis was performed on a Agilent 7900 quadrupole ICP-MS by the Centre for Trace Element Analysis, University of Otago. IR spectroscopy was performed directly on samples with a Bruker Alpha with ATR accessory. ^1^H and ^31^P NMR spectra of patch extracts were collected on a Varian 400 MR spectrometer. Solid state ^13^C NMR collected on a Bruker Avance III fitted with a 4 mm MAS BB probe. TGA was performed using a TA Q50 analyser with temperature ramp 20°C/minute. For X-ray diffraction studies*,* samples were tested as received and mounted in an aluminium sample holder. X-ray diffraction was conducted on a PANalytical X’Pert PRO MPD PW3040/60 diffractometer with a CuK_α_ source (λ=1.5406Å).  Continuous scan data were collected at diffraction angles between 3˚ and 90˚ operating at 40 kV and 30 mA with a step size of 0.008˚ and 4.03 seconds per step. Analysis and data processing was performed with X’Pert HighScore v4.0 software.

*Additive availability/release methodology*

D_2_O (1.4 mL) was added to a portion of the foam analyte (7.5 mg) and shaken for 30 s, then a 0.7 mL aliquot withdrawn for study via ^1^H NMR (PEO, F127, calcium acetate additives) or ^31^P NMR (polyphos).

*Additional characterisation*

IR spectroscopy showed characteristic, but often poorly resolved bands as expected for a naturally derived, polydisperse and moderately hygroscopic carbohydrate. Amide I and II bands at *circa* 1650 cm^-1^ and 1550 cm^-1^ respectively, with minimal differences between the raw and digested squid pen chitin, and the chitin patch. The 1150-900 cm^-1^ region was dominated by carbohydrate skeletal ether/alcohol C-O bands. For the modified patches additional bands were observed consistent with presence of the modifier component.

Elemental analysis is not reported for the chitin and modified chitin patches as the calculated variations are less than that associated with the adventitious and bound water.

|  | **Na** | **Mg** | **Al** | **P** | **K** | **Ca** | **Fe** | **Cu** | **Zn** |
| --- | --- | --- | --- | --- | --- | --- | --- | --- | --- |
|  | **(mg/kg)** | **(mg/kg)** | **(mg/kg)** | **(mg/kg)** | **(mg/kg)** | **(mg/kg)** | **(mg/kg)** | **(mg/kg)** | **(mg/kg)** |
| Squid pen | 6650 | 245 | 4.2 | 120 | 43 | 2250 | 15 | 25 | 240 |
| Chitin | 28 | 230 | 10.5 | 87 | 4.6 | 1900 | 18 | 3.9 | 21 |
| Detection limit | **2.00** | **1.00** | **0.10** | **5.00** | **2.00** | **4.00** | **0.20** | **0.01** | **0.10** |

**Supplementary Table**: Selected mineral component analysis from ICPMS

**Supplementary Figure 1.** IR spectra of squid pen, digested chitin and chitin patch.

**Supplementary Figure 2.** TGA of digested chitin and chitin patch.

*PC12 cell passage for cytotoxicity experiment:*

The cytotoxicity experiment requires seeding 96 well plates with 1 x 10^4^ cells per well. In order to ensure adequate number of cells were present for this experiment, multiple flasks of PC-12 cells were cultured until 80% confluence, prior to commencement. 3mL of trypsin was added to the flasks and incubated for 3 minutes, to ensure adequate detachment of the cells from the flasks. Following this step, 7mL of Dulbecco’s Modified Eagle Medium (DMEM) was added to the flasks and passed through pipette 3 times to resuspend the cells and break up clumps. The medium was subsequently aspirated from the flasks, and into a 50mL tube and centrifuged at 120xg for 3 minutes. The supernatant is removed from the tube and the cells are resuspended in DMEM. 20uL of cells were taken from the sample and mixed with 20uL of Trypan blue for cell counting and depending on the cell concentration, the cell sample is diluted to 1x10^4^ cells/100uL using DMEM. 100uL of the sample is then added to each well of the 96 well plate and incubated overnight at 37^o^C in a 5% CO_2_ in air atmosphere.
